# Toll-like receptor gene polymorphisms are associated with allergic rhinitis: a case control study

**DOI:** 10.1186/1471-2350-13-66

**Published:** 2012-08-02

**Authors:** Daniel Nilsson, Anand Kumar Andiappan, Christer Halldén, Wang De Yun, Torbjörn Säll, Chew Fook Tim, Lars-Olaf Cardell

**Affiliations:** 1 Division of ENT Diseases, Department of Clinical Sciences, Intervention and Technology, Karolinska Institutet, Stockholm, Sweden; 2 Biomedicine, Kristianstad University, Kristianstad, Sweden; 3 Department of Biological Sciences, National University of Singapore, Science Drive 4, Singapore, Singapore, 117543; 4 Department of Laboratory medicine, Lund University, Skåne University Hospital, Malmö, Sweden; 5 Department of Otolaryngology, National University of Singapore, 10 Lower Kent Ridge Road, Singapore, Singapore, 119260; 6 Department of Cell and Organism Biology, Lund University, Lund, Sweden

**Keywords:** Allergic rhinitis, Toll-like receptor, Polymorphism, Genetics, Haplotype, Case–control

## Abstract

**Background:**

The Toll-like receptor proteins are important in host defense and initiation of the innate and adaptive immune responses. A number of studies have identified associations between genetic variation in the Toll-like receptor genes and allergic disorders such as asthma and allergic rhinitis. The present study aim to search for genetic variation associated with allergic rhinitis in the Toll-like receptor genes.

**Methods:**

A first association analysis genotyped 73 SNPs in 182 cases and 378 controls from a Swedish population. Based on these results an additional 24 SNPs were analyzed in one Swedish population with 352 cases and 709 controls and one Chinese population with 948 cases and 580 controls.

**Results:**

The first association analysis identified 4 allergic rhinitis-associated SNPs in the *TLR7-TLR8* gene region. Subsequent analysis of 24 SNPs from this region identified 7 and 5 significant SNPs from the Swedish and Chinese populations, respectively. The corresponding risk-associated haplotypes are significant after Bonferroni correction and are the most common haplotypes in both populations. The associations are primarily detected in females in the Swedish population, whereas it is seen in males in the Chinese population. Further independent support for the involvement of this region in allergic rhinitis was obtained from quantitative skin prick test data generated in both populations.

**Conclusions:**

Haplotypes in the *TLR7-TLR8* gene region were associated with allergic rhinitis in one Swedish and one Chinese population. Since this region has earlier been associated with asthma and allergic rhinitis in a Danish linkage study this speaks strongly in favour of this region being truly involved in the development of this disease.

## Background

The human Toll-like receptor (TLR) proteins are pattern recognition proteins important in early detection of pathogens and subsequent triggering of the innate immune response [1]. All TLRs share the same basic organization with an extra-cellular domain containing a variable number of leucine-rich repeats, a single transmembrane spanning domain and an intracellular Toll-Interleukin-1 receptor (TIR) domain. TLRs are expressed on various cells of the immune system, including dendritic cells, macrophages and B- and T-cells. Some of the TLRs are located on the surface of the cell (TLR1, TLR2, TLR4, TLR5 and TLR6) while others are located intra-cellularly (TLR3, TLR7, TLR8 and TLR9). The different TLRs recognize different microbial products including: LPS (TLR4), viral dsRNA (TLR3), viral ssRNA (TLR7 and TLR8) and CpG DNA (TLR9) and some of the TLRs can even recognize several unrelated ligands. Homo- and hetero-dimerization of TLRs or association with other receptors such as CD14 increase the diversity of molecules that can be recognized by TLRs. Following encounter with a pathogen, TLRs trigger signalling cascades and the production of cytokines and chemokines but also interact with the adaptive immune response [1].

The 10 TLR genes are located on chromosomes 3, 4, 9 and X in a total of 6 chromosome regions. They are highly polymorphic with a large number of non-synonymous polymorphisms, some present at high frequencies [2]. Many studies have reported that genetic variation in the TLR genes modifies cellular immune response and alters susceptibility to disease [3]. Common polymorphisms primarily in *TLR2* and *TLR4*, but also polymorphisms in *TLR1TLR5TLR6TLR9* and *TLR10* have been associated with susceptibility to different infections. Although there is convincing evidence for a number of these findings, many studies rely on small sample sizes and have not been convincingly replicated [3]. A number of studies have searched for associations between genetic variation in the TLR genes and allergic disorders such as asthma, atopic eczema and allergic rhinitis (AR). Genetic variation in *TLR2*[4], *TLR4*[5], *TLR10*[6] and *TLR1TLR6* and *TLR10*[7] have been associated with the development of asthma, and in *TLR9* a promoter polymorphism has been associated with atopic eczema [8]. Other studies, however, have failed to find associations with genetic variants in *TLR2* and *TLR4* in atopic eczema [9] and in AR [10]. Linkage analysis identified a susceptibility locus on chromosome Xp for various atopic phenotypes including AR [11]. Since this chromosome region harbours the *TLR7* and *TLR8* genes, a later study investigated the possible role of these genes in the development of atopic disease [12]. SNPs in both genes showed significant associations with asthma, atopic dermatitis and AR.

Since TLRs are so centrally involved in the recognition of microbes and the initiation of the innate and adaptive immune responses, genetic changes in the TLR molecules may have profound effects on the development and severity of allergy. In addition, since a number of studies have reported significant associations between polymorphisms in the TLR genes and different atopic phenotypes we decided to search for associations with AR in these genes.

## Methods

### Ethics Statement

This study was approved by the Ethics Committee of the Medical Faculty, Lund University and the Institutional Review Board of National University of Singapore and written informed consent was obtained from all subjects. This study is also in compliance with the Helsinki declaration.

### Subjects and phenotypes

The Swedish study population was recruited at Malmö University hospital (Malmö, Sweden) between the years 2003 and 2009 and consists of unrelated subjects from the general population. It is comprised of 360 AR cases (169 females, 191 males, mean age 33 years). All cases were patients at the allergy clinic and were diagnosed with symptomatic birch and/or timothy grass pollen induced intermittent AR and 720 controls (294 females, 426 males, mean age 43 years) with no atopy and allergic symptomology. Both cases and controls were of Caucasian origin, with both parents born in Sweden. In the Swedish population skin prick tests (SPT) [13] were performed with a standard panel of 11 common airborne allergens (ALK-Abelló, Hørsholm, Denmark). This study population has previously been analyzed in several AR studies [14,15]. The Singapore Chinese population was collected in Singapore over multiple volunteer recruitment drives and consists of unrelated subjects. In the Singapore Chinese population SPT was using a panel consisting of common allergens in Singapore such as *Dermatophagoides pteronyssinus* and *Blomia tropicalis*. The population used in this study consists of 1024 AR cases (549 females, 475 males, mean age 22 years) with symptomatic dust mite induced AR and 605 controls (449 females, 156 males, mean age 22 years) with no atopy and allergic symptomology. This study population has previously been analyzed in several AR studies [16-19]. Diagnostic procedures for the study populations included personal interview of medical history and skin prick test (SPT) [13] or Phadiatop test and were performed using standard panels of common airborne allergens. SPT were performed on the volar side of the forearm with saline buffer as negative and histamine chloride (10 mg/ml) as positive controls. A wheal reaction diameter of ≥3 mm was considered a positive SPT response. SPT was only performed if the AR cases had not taken any anti-allergic drugs for at least 3 days prior to the test. Atopy is defined as a positive SPT reaction to either one of allergens tested. AR is thus diagnosed based on the presence of atopic status and typical AR symptoms as defined by the Allergic Rhinitis Impact on Asthma (ARIA) guidelines i.e., two or more AR symptoms (nasal congestion, rhinorrhea, nasal itching, sneezing) persisting for four or more days per week during the past year [20,21]. Conversely, the non-allergic controls are defined by having no atopy and no typical AR symptoms.

### Genotyping

Genomic DNA was extracted from blood collected in EDTA using QIAamp DNA Blood Maxi or Mini kits (Qiagen, Hilden, Germany) and DNA concentrations determined by fluorometry using PicoGreen (Molecular Probes, Invitrogen, Eugene, OR, USA). Genotypes were determined using the Sequenom MassARRAY MALDI-TOF system. Assay design was made using the MassARRAY Assay Design ver. 2.0 software (Sequenom Inc, San Diego, CA, USA) and primers were obtained from Metabion GmbH (Martinsried, Germany). HapMap (release 24) data were used (*r*^*2*^ cut off = 0.8, and minor allele frequency cut off = 0.2) to identify haplotype-tagging SNPs for each of the 10 TLR genes (*TLR1-TLR10*). In addition, non-synonymous SNPs reported in dbSNP or HapMap with minor allele frequencies above 5 % were added to this selection. Based on the results obtained in the first experiment, additional SNPs covering the *TLR7-TLR8* region were selected together with two SNPs reported in the literature as being associated with AR (rs179008 and rs2407992) [12]. All SNPs attempted and successfully genotyped are summarized and described in Additional file 1 and Additional file 2. A detailed description of genotyping and association testing in the two populations is given in Additional file 3.

### Statistical analysis

Statistical analyses were made using R statistical software [22] and the genetics package [23]. Genotype frequencies were calculated and tested for Hardy-Weinberg equilibrium in both cases and controls. Allele and genotype frequencies were then investigated for association with AR using a *χ*^*2*^-homogeneity test. The Kruskal-Wallis rank sum test was used for analysis of association between genotype and SPT-score. The score is defined as the size of the wheel reaction in relation to histamine. Haplotypes, haplotype blocks and linkage disequilibrium plots were constructed using Haploview 4.2 [24] and the default algorithm proposed by Gabriel *et al.*[25]. In order to compensate for the effect of multiple testing, the *q*-value introduced by Storey [26] was calculated using the software QVALUE (ver.1.0). The *q*-value is not just a modified *P*-value, as in the case of the Bonferroni correction, but is a conservative estimate of the false discovery rate given a *P*-value considered significant. *q*-values are reported if the corresponding uncorrected *P*-values are <0.05. Since the calculation of q-values is based on the distribution of *P*-values, it is not suited for significance testing of haplotypes. Instead the Bonferroni correction was used.

## Results

### Association testing *TLR1-TLR10* in the Swedish population

In a first exploratory step, tagSNPs were selected from HapMap data for each of the 10 TLR genes (*TLR1-TLR10*) and non-synonymous SNPs with reported frequencies in dbSNP or HapMap were added to this selection. A total of 73 SNPs were successfully genotyped in a subset of the Swedish population and subsequently tested for association with AR in 182 cases and 378 controls. Five of the analyzed genes contained no significant SNPs in the association analysis, whereas the genes *TLR1TLR6* and *TLR7* each showed one SNP, and *TLR8* had three SNPs with uncorrected *P*-values below 0.05 (Additional file 4). In none of these cases the *q*-values were below 0.1 giving only weak support for an association to allergy. However, the *TLR7* and *TLR8* genes were *a priory* implicated [12]. Given this fact, it is noteworthy that: 1) the three lowest *P*-values (0.02-0.04) and the three highest odds ratios (1.4-1.6) were found for SNPs in *TLR8*, 2) one additional SNP with a *P*-value below 0.05 was found in *TLR7*, and 3) the *TLR7* and *TLR8* genes are located in the same chromosome region. We therefore decided to extend the analysis of the *TLR7-TLR8* gene region. *TLR1* and *TLR6* which each showed a single marginally significant SNP and had no prior indication of association with AR were not further analyzed in the present study.

### Association testing the *TLR7-TLR8* region in the extended Swedish population

The *TLR7-TLR8* gene region was further investigated using a set of 24 SNPs. This set included the two SNPs previously associated with AR (rs179008 and rs2407992) by Møller-Larsen *et al.*[12]. All SNPs were genotyped in the complete Swedish population and subsequently tested for association with AR in 352 cases and 709 controls. The association analysis detected a total of 7 SNPs with *P*-values <0.05 at the genotype level. Three of these also showed *P*-values <0.05 in the allele test in females and in males - females combined (Table 1). In all cases the major allele was 6–7 % more common in cases compared with controls (Additional file 5). The genotype tests gave *q*-values equal to 0.06 for all 7 SNPs. Two SNPs gave slightly lower *q*-values (0.04) for the allele test in females. None of the seven SNPs showed a deviation from the Hardy-Weinberg equilibrium (Table 1).

**Table 1:** **Association of SNPs in the *TLR7-TLR8* region with AR phenotype in the Swedish population**

						**Association test**				**Skin prick test**	
**Gene**	**SNP ID**	Position^*^	**Alleles**	**MAF**^†^	**HW**	**Allele M + F**	**Allele M**	**Allele F**	**Genotype**	**Birch**	**Timothy grass**
*TLR7*	rs179021	12889763	T/G	0.22	0.25	0.60	0.54	0.82	0.50	0.64	0.061
*TLR7*	rs179020	12889857	G/A	0.24	0.32	0.40	0.71	0.16	0.36	0.17	0.49
*TLR7*	rs179019	12889969	C/A	0.24	0.32	0.41	0.69	0.16	0.36	0.17	0.49
*TLR7*	rs179016	12894442	G/C	0.36	0.52	0.34	0.31	0.69	0.61	0.90	0.14
*TLR7*	rs179014	12899765	G/A	0.24	0.13	0.58	0.65	0.25	0.45	0.19	0.43
*TLR7*	rs179013	12901471	G/A	0.22	0.44	0.42	0.50	0.56	0.63	0.42	0.095
*TLR7*	rs179012	12901562	G/A	0.29	0.98	0.67	0.68	0.84	0.83	0.32	0.28
*TLR7*	rs179011	12901960	G/T	0.23	0.20	0.41	0.63	0.44	0.57	0.46	0.093
*TLR7*	rs179010	12902885	C/T	0.31	0.012	0.47	0.95	0.26	0.55	0.97	0.90
*TLR7*	rs179008	12903659	A/T	0.22	0.16	0.29	0.38	0.47	0.44	0.39	0.11
*TLR7*	rs864058	12906030	G/A	0.07	0.80	0.37	0.29	0.65	0.56	0.44	0.44
*TLR7*	rs179007	12910322	A/G	0.23	0.81	0.53	0.50	0.90	0.87	0.20	0.21
*TLR7*	rs179006	12910521	G/A	0.20	0.61	0.17	0.46	0.30	0.43	0.42	0.22
*TLR7*	rs2269809	12910619	G/T	0.50	0.057	0.58	0.91	0.40	0.028 (0.064)	0.81	0.054
*TLR7*	rs5935438	12913022	G/C	0.45	0.21	0.28	0.99	0.22	0.0059 (0.064)	0.81	0.019 (0.051)
*TLR8*	rs178998	12917787	C/T	0.34	0.045	0.19	0.86	0.080	0.15	0.10	0.23
*TLR8*	rs3788935	12922659	A/G	0.23	0.53	0.0036 (0.076)	0.36	0.0037 (0.046)	0.012 (0.064)	0.44	0.23
*TLR8*	rs3761624	12923681	A/G	0.22	0.62	0.0080 (0.076)	0.49	0.0056 (0.046)	0.016 (0.064)	0.40	0.16
*TLR8*	rs5741883	12924221	C/T	0.25	0.96	0.28	0.96	0.17	0.068	0.67	0.038 (0.054)
*TLR8*	rs17256081	12926045	T/C	0.49	0.11	0.80	0.64	0.98	0.021 (0.064)	0.76	0.0066 (0.035)
*TLR8*	rs4830805	12927759	G/A	0.21	0.92	0.0069 (0.076)	0.25	0.013 (0.071)	0.042 (0.064)	0.34	0.080
*TLR8*	rs1548731	12927947	C/T	0.27	0.93	0.50	0.89	0.48	0.046 (0.064)	0.94	0.49
*TLR8*	rs4830808	12932334	C/T	0.16	0.44	0.70	0.53	0.38	0.55	0.24	0.22
*TLR8*	rs2407992	12939112	G/C	0.38	0.068	0.63	0.75	0.40	0.67	0.42	0.040 (0.054)

The Hardy-Weinberg (HW) test uses both cases and controls. Male (M) and Female (F) individuals were used separately and combined in the allele tests. The Kruskal-Wallis test is used on skin prick data. *P*-values are given for the HW and the association tests. *Q*-values are given in parenthesis.

^*^Position according to NCBI build 37.1. ^†^Allele frequency of second allele.

Using Haploview on HapMap CEU data to analyze the haploblock structure of the *TLR7-TLR8* region identifies 3 haploblocks; one encompassing the promoter region and a major part of the *TLR7* gene, one covering the last part of *TLR8* and one encompassing the region in between those two blocks (Additional file 6). The 7 indicated SNPs are all located in the latter haploblock. Haplotypes were constructed from the 3 SNPs that were associated both at the allele and genotype levels (rs3788935, rs3761624 and rs4830805) and had the highest odds ratios (Additional file 5: Table S4). When analyzing males and females together, the most common haplotype (AAG) showed a higher frequency in cases than in controls, 0.81 versus 0.75. This difference was significant (*P* = 0.0045, uncorrected *P*-value) and compatible with the notion of this haplotype being a risk-haplotype (Table 2). This was true also when analyzing females separately (*P* = 0.0061), but when analyzing males, none of the haplotypes showed any association. Given the data in Table 2, a Bonferroni correction for nine tests is the most conservative approach. This gives a *P*-value limit at 0.0055, rendering the haplotype test for all individuals still significant (*P* = 0.0045).

**Table 2:** **Association of haplotypes in *TLR8* with AR in the Swedish population**

	**SNP ID**			**Haplotype frequency**		**Association test**		
**Haplotype**	**rs3788935**	**rs3761624**	**rs4830805**	**709 controls**	**352 cases**	**M + F**	**M**	**F**
H1	**A**	**A**	**G**	0.75	0.81	0.0045	0.32	0.0061
H2	G	G	A	0.22	0.17	0.0092	0.31	0.014
H3	G	G	**G**	0.025	0.019	0.48	0.80	0.26

Male (M) and female (F) individuals were used separately and combined in the association tests. Haplotype

frequencies are given as the combined frequencies for males and females. Disease-associated alleles are in bold.

To test for genotype effects on the level of AR among the cases the Kruskal-Wallis test was used. The SPT scores for birch and timothy grass were tested against all 24 SNPs in the *TLR7-TLR8* region. A total of 4 SNPs had *P*-values <0.05when analyzing timothy grass SPT data (Table 1), including the rs2407992 SNP previously associated with AR by Møller-Larsen *et al.*[12]. The strongest association was found for rs17256081 (*P* = 0.0066 and *q* = 0.03). The remaining three indicated SNPs had *q*-values equal to 0.05. If the effect of genotype on SPT scores is investigated in detail for this SNP, it is seen that the heterozygotes have a lower average score than the homo- and hemizygotes. The same general effect is seen in rs5935438 and rs2407992. No SNPs were detected as significant when birch SPT data were analyzed.

### Association testing the *TLR7-TLR8* region in the Chinese population

The same set of SNPs was also genotyped and 23 SNPs were subsequently analyzed for association with AR in a Chinese population containing 948 cases and 580 controls. The association analysis detected a total of 5 SNPs with *P*-values below 0.05 when males were analyzed separately; the *q*-values for these five SNPs were 0.07. All but one of the indicated SNPs had genotype distributions that were in Hardy-Weinberg equilibrium (Table 3). The rs2407992 SNP showed the strongest association in the Chinese population (*P* = 0.0054) with the major allele 10 % more common among male cases than male controls (Additional file 7).

**Table 3:** **Association of SNPs in the *TLR7-TLR8* region with AR phenotype in the Chinese population**

						**Association test**				**Skin prick test**	
**Gene**	**SNP ID**	Position^*^	**Alleles**	**MAF**^†^	**HW**	**Allele M + F**	**Allele M**	**Allele F**	**Genotype**	** *B. tp* **^‡^	** *D. pt* **^*#*^
*TLR7*	rs179021	12889763	T/G	0.01	1.0	0.72	0.42	0.62	0.62	0.70	0.12
*TLR7*	rs179020	12889857	G/A	0.25	0.26	0.65	0.38	0.36	0.56	0.021 (0.32)	0.010 (0.025)
*TLR7*	rs179019	12889969	C/A	0.25	0.34	0.78	0.36	0.44	0.70	0.022 (0.32)	0.0090 (0.025)
*TLR7*	rs179016	12894442	G/C	0.12	1.0	0.61	0.62	0.81	0.85	0.094	0.013 (0.025)
*TLR7*	rs179014	12899765	G/A	0.00	1.0	0.80	-	0.91	0.91	0.091	0.24
*TLR7*	rs179013	12901471	G/A	0.01	1.0	0.85	0.42	0.76	0.76	0.72	0.14
*TLR7*	rs179012	12901562	G/A	0.06	1.0	0.66	0.92	0.75	0.95	0.67	0.011 (0.025)
*TLR7*	rs179011	12901960	G/T	0.01	1.0	0.65	0.42	0.58	0.58	0.73	0.13
*TLR7*	rs179010	12902885	C/T	0.35	0.18	0.86	0.45	0.52	0.59	0.078	0.0046 (0.025)
*TLR7*	rs179008	12903659	A/T	0.00	1.0	0.37	-	0.47	0.47	0.76	0.43
*TLR7*	rs864058	12906030	G/A	0.00	0.0091	0.92	0.57	0.85	0.39	0.28	0.88
*TLR7*	rs179007	12910322	A/G	0.00	0.0044	0.17	-	0.25	0.55	0.76	0.43
*TLR7*	rs179006	12910521	G/A	0.00	0.0036	0.17	-	0.25	0.55	0.76	0.43
*TLR7*	rs2269809	12910619	T/G	0.16	0.20	0.38	0.061	0.97	0.27	0.59	0.18
*TLR7*	rs5935438	12913022	G/C	0.15	0.017	0.34	0.056	0.92	0.27	0.77	0.26
*TLR8*	rs178998	12917787	T/C	0.16	0.053	0.18	0.024 (0.072)	0.74	0.34	0.43	0.56
*TLR8*	rs3788935	12922659	G/A	0.17	0.10	0.27	0.025 (0.072)	0.85	0.80	0.45	0.14
*TLR8*	rs5741883	12924221	C/T	0.02	0.35	0.66	0.84	0.64	0.39	0.27	0.53
*TLR8*	rs17256081	12926045	T/C	0.15	0.018	0.20	0.010 (0.072)	0.86	0.54	0.84	0.19
*TLR8*	rs4830805	12927759	A/G	0.19	0.29	0.42	0.016 (0.072)	0.84	0.74	0.40	0.053
*TLR8*	rs1548731	12927947	C/T	0.02	0.68	0.33	0.93	0.38	0.26	0.16	0.25
*TLR8*	rs4830808	12932334	T/C	0.23	0.41	0.16	0.054	0.58	0.85	0.091	0.057
*TLR8*	rs2407992	12939112	C/G	0.19	0.18	0.023 (0.33)	0.0054 (0.072)	0.30	0.59	0.12	0.45

The Hardy-Weinberg (HW) test uses both cases and controls. Male (M) and female (F) individuals were used separately and combined in the allele tests. The Kruskal-Wallis test is used on skin prick data. *P*-values are given for the HW and association tests.

^*^Position according to NCBI build 37.1.

^†^Allele frequency of second allele.

^‡^*Blomia tropicalis.*

^#^*Dermatophagoides pteronyssinus.*

The LD pattern for the *TLR7-TLR8* region in the Chinese population was investigated using Haploview and HapMap CHB data. The analysis revealed one haploblock spanning the major part of *TLR7* and one haploblock spanning the last part of *TLR7*, the intergenic region and the complete *TLR8* gene (Additional file 6). Haplotypes were constructed from the 5 SNPs that were indicated to be associated with AR (i.e. rs178998, rs3788935, rs17256081, rs4830805 and rs2407992; Table 4). These SNPs are all located in the haploblock covering the *TLR8* gene. The most common haplotype (TGTAC) showed a higher frequency in cases than in controls, 0.76 versus 0.73, which was only marginally significant (*P* = 0.044). When analyzing females separately, the frequencies of this haplotype did not differ between cases and controls (*P* = 0.53), but when comparing frequencies among male cases and controls, 0.80 versus 0.66, the difference is significant (*P* = 0.0016). Given the data in Table 4, a Bonferroni correction for 20 tests is the most conservative approach. This gives a *P*-value limit at 0.0025, rendering the haplotype test for males still significant. Of the five SNPs listed above, two were also included in the haplotype analysis in the Swedish population (rs3788935 and rs4830805). For both SNPs, the associated alleles are the major alleles in both populations. Since the allele frequencies of the two populations are highly different the opposite alleles are associated with AR in the two populations. This is illustrated for rs3788935 where the two alleles, A and G, have frequencies 0.77 and 0.23 in the Swedish population (Table 1) and 0.17 and 0.83 in the Chinese population (Table 3). Since LD is relatively strong in this region the same scenario is seen for a number of SNPs in the region.

**Table 4:** **Association of haplotypes in *TLR8* with AR in the Chinese population**

	**SNP ID**					**Haplotype frequency**		**Association test**		
**Haplotype**	**rs178998**	**rs3788935**	**rs17256081**	**rs4830805**	**rs2407992**	**580 controls**	**948 cases**	**M + F**	**M**	**F**
H1	**T**	**G**	**T**	**A**	**C**	0.73	0.76	0.044	0.0016	0.53
H2	C	A	C	G	G	0.12	0.11	0.34	0.070	0.82
H3	**T**	**G**	**T**	**A**	G	0.064	0.047	0.065	0.30	0.19
H4	C	A	C	G	**C**	0.025	0.021	0.45	0.37	0.73
H5	**T**	A	**T**	G	**C**	0.017	0.021	0.48	0.70	0.41
H6	**T**	**G**	**T**	G	**C**	0.012	0.014	0.69	0.80	0.58
H7	C	**G**	**T**	**A**	G	0.012	0.010	0.55	-	0.72

Male (M) and female (F) individuals were used separately and combined in the association test. Haplotype frequencies are given as the combined frequencies for males and females. Disease-associated alleles are in bold.

The Kruskal-Wallis test was used to determine the effect of genotype on the level of allergy of *Dermatophagoides pteronyssinus* and *Blomia tropicalis* that are the two major allergens in Singapore. Five SNPs located in the *TLR7* gene showed uncorrected *P*-values below 0.05 and *q*-values equal to 0.025 for an SPT response for *D. pteronyssinus* between genotypes. Two of those (rs179020 and rs179019) had *P*-values <0.05 when testing the response for *B. tropicalis*, however both of these had high *q*-values and are thus not indicated (Table 3). None of the SNPs showed significant differences when analyzing females and males separately.

## Discussion

A number of observations indicate that genetic variation in the *TLR7TLR8* gene region influences the risk for and the degree of AR: 1) The *a priori* indicated *TLR7* and *TLR8* genes gave the strongest signals among the tested TLR genes. 2) Association tests in both the Swedish and the Chinese populations yielded several uncorrected p-values <0.05 with their corresponding q-values <0.1 and in some cases <0.05. When haplotype associations were tested using the Bonferroni correction, significant differences were still observed. 3) Comparing the results from the Swedish and Danish populations, a similar pattern of association was seen. In the Danish population [12], two SNPs were associated with AR, rs179008 in *TLR7* and rs2407992 in *TLR8*. The A-allele of the rs179008 SNP was over-transmitted in Danish cases (*P* = 0.0039). This pattern was seen also in the Swedish population, albeit at a non-significant level (*P* = 0.38). In the same way, the G-allele of the rs2407992 SNP was over-transmitted in Danish cases (*P* = 0.037) with the same pattern observed in Swedish cases, but again at a non-significant level (*P* = 0.40). Thus, the congruence of the allelic associations detected in the Swedish and Danish populations may simply reflect the fact that they have the same major allele. 4) When the effect of genotype on the severity of phenotype among the cases were tested, several p-values yielded uncorrected p-values <0.05 and corresponding q-values < 0.1 and in some cases < 0.05. This is particularly important since the phenotype test relies on cases only and furthermore do not depend on the exact genotype numbers among the cases. Thus, the phenotype test is independent from the case–control association test. In addition, a recent genome wide association study for AR based on genotyping using the Illumina HumanHap 550 k BeadChip observed no SNPs with genome wide significance, and only a few SNPs showing suggestive association in *MRPL4, BCAP, CSF1R* and *DNAJC6*[18]. In concordance with the results of the present study, none of the TLR-genes of the autosomes showed suggestive associations. Since the GWAS did not present data on SNPs from the X and Y chromosomes, no data are available for TLR7 and TLR8 and the results of the two studies cannot be directly compared for these two genes.

Although a number of results indicate an association between genetic variation in *TLR7* and *TLR8* and occurrence and degree of AR, two discordant observations have been made. The first is that, for many of the SNPs in the region, the major allele in the Swedish population is the minor allele in the Chinese population and vice verse. However, in both populations it is the most common haplotype that is associated with AR. A situation where different major alleles of the same locus are risk-associated in different populations is compatible with a scenario where the risk is contributed by many different rare alleles that each has a relatively limited life span. Under these conditions, the major haplotypes will on average be more associated with risk. There are good reasons to believe that multiple rare variants, both within and across genes, collectively affect the expression and function of genes. This hypothesis can be tested by simply re-sequencing large numbers of cases and controls and evaluate the patterns of rare alleles in the two groups. Many reports on the association of rare variants with specific disease phenotypes exist (reviewed in Bansal *et al.*[27]).

The second discordant observation is that, in the Swedish population the AR association is seen primarily in females, whereas in the Chinese population the association is observed among males only. However, the Swedish population is most likely underpowered when analyzing X-chromosomes in males and a contribution to disease from males can therefore not be ruled out. In fact, when analyzing both males and females together, the *P*-values of the significant SNPs (rs3788935, rs3761624, rs4830805) are almost the same compared to when analyzing females separately. Also, when analyzing males, a tendency for association among these SNPs (*P*-values from 0.25 to 0.49) can be observed. In the Chinese population, however, the observed association is restricted to males with no tendency of association among females. Also in the study by Møller-Larsen *et al.*[12] several strong sex-specific differences were identified.

A number of studies have reported associations between various diseases and variants in the *TLR7* or *TLR8* genes. For example, systemic lupus erythematosus was found to be associated with variation in *TLR7*[28] and Crohn’s disease and ulcerative colitis with variation in *TLR8*[29]. In particular, the only high frequency missense polymorphism in the *TLR8* gene (rs3764880) has been implicated in a number of diseases, such as tuberculosis [30] and progression of HIV infection [31]. This SNP is in perfect LD with rs3761624, indicating that these SNPs may serve as proxies for each other. The common allele of rs3761624 (A-allele in the CEU population) is present on the risk haplotype detected in the Swedish population, being compatible with the reported disease associations for rs3764880 where the common allele is the disease-associated allele. The pleiotropic effect seen for this allele also supports the disease association in the present study. Just as in the case of AR in the present study, several of the studies cited above reveal sex-specific disease associations for the *TLR7-TLR8* gene region. In the association study of Crohn’s disease and ulcerative colitis one risk- and one protective haplotype was detected among females, and the study of systemic lupus erythematosus detected an association to this region with a stronger effect in males compared to females (OR, male/female = 2.3, 95 % CI = 1.64-3.30).

TLR7 is expressed by inflammatory and structural cells in both the upper and lower airways. It has a well-defined protective role during viral infection. We have previously demonstrated that TLR7- mediated activation of eosinophils is related to the atopic status of the patient and that the presence of a Th2-like cytokine milieu affects the outcome of the response [32]. Thus, eosinophil activation via TLR7 might engender a link between viral infection and allergic exacerbations. Others have, using various animal models, demonstrated beneficial effects of TLR7 agonists in allergic asthma [33] and a recent clinical trial has shown encouraging results, suggesting that TLR7 agonists might be a novel alternative for treatment of allergic rhinitis [34].

Association analysis based on case–control populations is a very popular method to search for genes that influence specific phenotypes. However, many studies suffer from limited population sizes, where the obvious drawback is the risk of false positives. This emphasizes the importance of large population sizes and the replication of positive findings but also of meta-studies where the combined efforts of many studies are evaluated. In the present study, genetic variation in the *TLR7-TLR8* gene region was associated with AR in one Swedish and one Chinese population. Since this region has earlier been associated with asthma and AR in a Danish linkage study and since TLR7 agonists have shown beneficial effects both in allergic asthma and in allergic rhinitis, this speaks strongly in favour of this region being truly involved in the development of AR.

## Conclusions

Haplotypes in the *TLR7-TLR8* gene region were associated with allergic rhinitis in one Swedish and one Chinese population. Since this region has earlier been associated with asthma and allergic rhinitis in a Danish linkage study this speaks strongly in favour of this region being truly involved in the development of this disease.

## Competing interest

The authors confirm that there are no conflicts of interest.

## Authors’ contribution

DN, CH and LOC designed the study. DN, TS and AKA performed the data analysis and DN, CH, TS and LOC wrote the manuscript. All authors critically revised the manuscript and approved the final form of the manuscript.

## Pre-publication history

The pre-publication history for this paper can be accessed here:

http://www.biomedcentral.com/1471-2350/13/66/prepub

## Supplementary Material

Additional file 1 Investigated genes and number of attempted and genotyped SNPs. Click here for file

Additional file 2 Description of genotyped SNPs. Click here for file

Additional file 3 Description of genotyping and association testing in the two populations. Click here for file

Additional file 4 Complete first experiment association test results in the Swedish population. Click here for file

Additional file 5 Allele and genotype frequencies for all significant SNPs in the Swedish population. Click here for file

Additional file 6 **Linkage disequilibrium plot of *TLR7-TLR8* gene region constructed using Haploview software and HapMap data (release 24) of the A) CEU population and B) CHB population.** The intensity of shading represents D´. ^S^ SNPs showing single-point association in the Swedish population. ^C^ SNPs showing single-point association in the Chinese population. ^D^ SNPs showing single-point association in Møller-Larsen *et al.* (2008). Click here for file

Additional file 7 Allele frequencies in Chinese males for all significant SNPs Click here for file
