# Supplementary material for: Toll-like receptor gene polymorphisms are associated with allergic rhinitis: a case control study
Source: BMC Med Genet. 2012 Aug 2;13:66. doi: 10.1186/1471-2350-13-66 (PMC3459792; doi:10.1186/1471-2350-13-66)
Supplement: Additional file 1 — Investigated genes and number of attempted and genotyped SNPs. [file 1471-2350-13-66-S1.pdf]

Table S1. Investigated genes and number of attempted and genotyped SNPs

Chromosome position data from NCBI database, GRCh 37.

Gene	Chromosome position	Size (kbp)	No. of attempted SNPs	No. of successfully genotyped SNPs
**Swedish population, first screen**				
TLR1	Chr 4 (38,797,876-38,806,412)	8.5	11	8
TLR2	Chr 4 (154,605,441-154,627,243)	21.8	8	8
TLR3	Chr 4 (186,990,309-187,006,252)	15.9	10	8
TLR4	Chr 9 (120,466,460-120,479,768)	13.3	12	7
TLR5	Chr 1 (223,283,584-223,316,624)	33	13	0
TLR6	Chr 4 (38,828,408-38,831,160)	2.8	10	7
TLR7	Chr X (12,885,202-12,908,480)	23.3	13	13
TLR8	Chr X (12,924,758-12,941,288)	16.5	15	10
TLR9	Chr 3 (52,255,098-52,260,179)	5.1	4	4
TLR10	Chr 4 (38,774,234-38,784,589)	10.4	11	8
**Swedish population, second screen**				
TLR7-TLR8	Chr X (12,885,202-12,941,288)	56.1	30	24
**Chinese population**				
TLR7-TLR8	Chr X (12,885,202-12,941,288)	56.1	30	23
