# Supplementary material for: Toll-like receptor gene polymorphisms are associated with allergic rhinitis: a case control study
Source: BMC Med Genet. 2012 Aug 2;13:66. doi: 10.1186/1471-2350-13-66 (PMC3459792; doi:10.1186/1471-2350-13-66)
Supplement: Additional file 2 — Description of genotyped SNPs. [file 1471-2350-13-66-S2.pdf]

Table S2. Description of genotyped SNPs

Chromosome positions are given as chromosome number followed by chromosome positions according to NCBI database, GRCh 37. Base changes are given as major/minor alleles from HapMap data, release 24. Minor allele frequencies are given for the HapMap CEU population, whereas the allele frequencies reported for the HapMap CHB population are given for the minor allele in the CEU population. Primer sequences are given in 5´-3´direction.

Exp.*	SNP ID	Chromosome position		Gene location		Function	Alleles	HapMap allele frequency		Primer sequences		
								CEU	CHB	Forward	Reverse	Extension
1	rs352140	3	52256697	*TLR9*	exon 2	cds-synonymous	C/T	0.49		ACGTTGGATGATAAGCTGGACCTCTACCAC	ACGTTGGATGTGGCTGTTGTAGCTGAGGTC	CACTCATTCACGGAGCTACC
1	rs352139	3	52258372	*TLR9*	intron		A/G	0.50		ACGTTGGATGGAAGATGCTAGAAGATGCCC	ACGTTGGATGTGGGAGGGCTGTGTGAGTG	GTGGAGTGGGTGGAGGT
1	rs5743836	3	52260782	*TLR9*	near-gene-5		C/T	-		ACGTTGGATGTTGGGATGTGCTGTTCCCTC	ACGTTGGATGAGCAGAGACATAATGGAGGC	CTGTTCCCTCTGCCTG
1	rs187084	3	52261031	*TLR9*	near-gene-5		T/C	0.35		ACGTTGGATGTATTCCCCTGCTGGAATGTC	ACGTTGGATGTGCTGGGCACTGTACTGGAT	CCCCTGCTGGAATGTCAGCTTCTT
1	rs10776483	4	38775040	*TLR10*	exon 4	cds-synonymous	T/C	0.29		ACGTTGGATGATGGAATGGGTTCCAGTAAG	ACGTTGGATGTGAATTCTACTTTGCCCACC	GGTTCCAGTAAGATAAGAATTAT
1	rs11096955	4	38776107	*TLR10*	exon 4	missense	A/C	0.40		ACGTTGGATGTGAGAGTTTTCAAGTGAGGC	ACGTTGGATGCCAATAATATCTTAACAGACG	AAGTGAGGCAGTTGGA
1	rs11096956	4	38776180	*TLR10*	exon 4	cds-synonymous	G/T	0.30		ACGTTGGATGCCTGACAATATCAAATGCAC	ACGTTGGATGAATATTGGAATTTCGTAGG	CCACACATGCTTTTCCC
1	rs11096957	4	38776491	*TLR10*	exon 4	missense	A/C	0.41		ACGTTGGATGTAAGCAATAGAACCGATGTC	ACGTTGGATGGGCAAAAGCCAATTTGTAAG	AAGCAATAGAACCGATGTCTTAGCAT
1	rs10856839	4	38777236	*TLR10*	exon 4	UTR-5	A/C	0.13		ACGTTGGATGGTTTCTGATGAGTCTCATTG	ACGTTGGATGGGGTTTTGAGCTCATCTTC	TCCAAGGATTTTACCACT
1	rs4274855	4	38777471	*TLR10*	exon 3	UTR-5	G/A	0.28		ACGTTGGATGTCCTCTCTGAGAATCCTGAC	ACGTTGGATGTTGGCTGAGAAGTCTCCAAG	AATCCTGACTTACCTCAACAAC
1	rs7694115	4	38779094	*TLR10*	intron	-	A/G	0.39		ACGTTGGATGCTATAGGTTGCCTCAAACAG	ACGTTGGATGCAGCAGAAGATTTAGAGTCC	AAATGTATGTCAGAACCTG
1	rs7698870	4	38781459	*TLR10*	intron	-	G/A	0.03		ACGTTGGATGTACCACGGGAATGAACAGAG	ACGTTGGATGGATCTCTTCTGAATGACCTC	GAACAGAGAAGGGACAGGTGA
1	rs4833095	4	38799710	*TLR1*	exon 4	missense	T/C	0.30		ACGTTGGATGCTGGAGGATCCTAATGAAAG	ACGTTGGATGCCTAAGTATTCTGGCGAAAC	GTTTCAATGTTGTTTAAGGTAAGA
1	rs5743596	4	38802528	*TLR1*	exon 3	UTR-5	C/T	0.24		ACGTTGGATGTGCTCAGGGTCTTCATGAAC	ACGTTGGATGGGTGCCCAATATGCCTTTGT	ACCAGGCCCTCTTCCT
1	rs5743595	4	38802644	*TLR1*	intron	-	T/C	0.28		ACGTTGGATGGGAAATTTCCGGGTCTTTCA	ACGTTGGATGGCAAGGAAGATGATGCAGAC	CGGGTCTTTCAGCCAAA
1	rs5743594	4	38802751	*TLR1*	intron	-	C/T	0.10		ACGTTGGATGGCTACTCACACAAGGAGCAA	ACGTTGGATGGGGTTAGGATTTCAACATAAG	GGATGTTATAGCTTGAATGTTT
1	rs5743580	4	38804405	*TLR1*	intron	-	A/G	-		ACGTTGGATGAACATCCAGAGTGACTCAGC	ACGTTGGATGTGGCTATGGTAAGCTTCTC	AGTGACTCAGCGAGTTTAGAG
1	rs5743566	4	38805942	*TLR1*	exon 2	UTR-5	C/G	-		ACGTTGGATGAAGGACTAGCTAGTGGGAAG	ACGTTGGATGAGTTACTCCCGGAGGCAATG	ACAAACCTGGCCACAAAAACA
1	rs5743565	4	38805983	*TLR1*	exon 2	UTR-5	A/G	0.26		ACGTTGGATGAGCTGAACAGCAGCATTGCC	ACGTTGGATGTTCTCTTCACCTAATCCCGC	CCGGGAGTAACTGACT
1	rs5743557	4	38806827	*TLR1*	near-gene-5		C/T	0.28		ACGTTGGATGGAGTGTGCTTCAGCAAAAC	ACGTTGGATGTCTTGCCTTTTCATCCATCC	ATCTCGACCCCTCCCTCTTT
1	rs5743818	4	38829163	*TLR6*	exon 1	cds-synonymous	G/T	-		ACGTTGGATGCATACCCTTAGAAGAACTCC	ACGTTGGATGCCAGGCAGAATCATGTTCAC	ACCTCCAGTTTCATGC
1	rs3821985	4	38830012	*TLR6*	exon 1	cds-synonymous	C/G	0.28		ACGTTGGATGAAAATACTATCTGTGAAAAC	ACGTTGGATGATGCTGTGTCCTCATGCACC	ACTATCTGTGAAAACGTTCTG
1	rs5743810	4	38830350	*TLR6*	exon 1	missense	C/T	0.42		ACGTTGGATGATTTTTATCAGAACTCACC	ACGTTGGATGAGGCATTTCCAAGTCGTTTC	AGAACTCACCAGAGGT
1	rs5743808	4	38830736	*TLR6*	exon 1	missense	T/C	0.00		ACGTTGGATGGTTGCAAAAGATATCCTGCC	ACGTTGGATGGCAGGGCCTTGAAATCATTG	AAGATATCCTGCCATCCTA
1	rs1039559	4	38831596	*TLR6*	near-gene-5		T/C	0.46		ACGTTGGATGGCAAGCAGCAGACACATCAA	ACGTTGGATGCTCAGCCTTTTTCTCCCAC	GGAAATATGCAAAGATATATGGA
1	rs5743805	4	38832059	*TLR6*	near-gene-5		C/T	-		ACGTTGGATGCCCCAAGTTCTGGGACTATA	ACGTTGGATGCAAGAAGCAACAATACACCC	GGCTTAAGCCACTGTGC
1	rs5743788	4	38833207	*TLR6*	near-gene-5		C/G	0.46		ACGTTGGATGGACACTGCAAGAGAGTGAAA	ACGTTGGATGCAAGTCCCTATCATATATGG	TGAAAAAGACAAACCACAGACT
1	rs893629	4	154604968	*TLR2*	near-gene-5		G/A	0.01		ACGTTGGATGACATCACCTGAAACACACCG	ACGTTGGATGGATTCATGAGCCTGAGTTCG	TCCACTCCTGATGCTT
1	rs4696480	4	154607126	*TLR2*	intron		T/A	0.00		ACGTTGGATGCTCACCATGTGATGCTTTCC	ACGTTGGATGGGGAAGTCCAAGATTGAAGG	TGTAGCCAGATGACCCTC
1	rs1898830	4	154608453	*TLR2*	intron		A/G	0.35		ACGTTGGATGGATCCCCTATTTTCTAGCAC	ACGTTGGATGAAAACTGGAAAAGGAATAG	CTTATATTATTATTTCCCCTGTTC
1	rs1816702	4	154609523	*TLR2*	intron		C/T	0.15		ACGTTGGATGGTGTGAGCCTTACTAAAGGT	ACGTTGGATGCTTTGATTCCTCTAGCGCTG	GTAACTTAGAATTACAATGGACTGC
1	rs4235232	4	154618084	*TLR2*	intron		T/G	0.01		ACGTTGGATGGCTGTTCTTACCCAAAACAC	ACGTTGGATGTGTGGTACTAGAATTACCAG	AAAACACATCTTACCTTCTTTTC
1	rs3804099	4	154624656	*TLR2*	exon 3	cds-synonymous	T/C	0.44		ACGTTGGATGCTGCTTCATATGAAGGATCAG	ACGTTGGATGGATCTACAGAGCTATGAGCC	CTTCATATGAAGGATCAGATGACTTAC
1	rs3804100	4	154625409	*TLR2*	exon 3	cds-synonymous	T/C	0.05		ACGTTGGATGTTGAACTTATCCAGCACACG	ACGTTGGATGTTCCAGTGTCTTGGGAATGC	AACTTATCCAGCACACGAATACACAG
1	rs5743704	4	154625951	*TLR2*	exon 3	missense	C/A	0.05		ACGTTGGATGTGCATCATAGCAGATGTTCC	ACGTTGGATGTGAAAATGATGTGGGCCTG	cGCTGGGAGCTTTCCTG
1	rs5743303	4	186988853	*TLR3*	near-gene-5		A/T	0.18		ACGTTGGATGGGGATAGGTATGAAGGATTG	ACGTTGGATGATTGGAATGGTGTCATATGC	gAGGATTGTTGAGATGATGTGTTTT
1	rs5743305	4	186989333	*TLR3*	near-gene-5		T/A	0.35		ACGTTGGATGATCAGAGACATGTAGCCCTG	ACGTTGGATGAAAGTGCCCTCTTGTGGGCT	TGAGCCCAGTAACTATAAAGCGG
1	rs7657186	4	186994039	*TLR3*	intron		G/A	0.20		ACGTTGGATGCTTCTCACTAGAATGTGAAGC	ACGTTGGATGTCTTGTTCTAGGCACAGAC	TAGAGGACATAGTTCTTATTCCATA
1	rs3775296	4	186997767	*TLR3*	exon 2	UTR-5	G/T	0.16		ACGTTGGATGGCCATCTGCTATTAATGTTGC	ACGTTGGATGTACAAGGCAAAGTCTGTCTC	ACTTTTTAATGTTTCTTTTCTACAG
1	rs5743312	4	187000256	*TLR3*	intron		C/T	0.16		ACGTTGGATGGTGGATAGTCCCTATCTGTG	ACGTTGGATGGGAAGGATTGCTGGAAGACA	TAGTCCCTATCTGTGTCACATACA
1	rs5743314	4	187000375	*TLR3*	intron		C/G	-		ACGTTGGATGTTCCACCCAGTGCTGCAGG	ACGTTGGATGTGGTGTCATCCTCCTGAGAG	TGCAGGGCGGCAGAGTCC
1	rs3775292	4	187003025	*TLR3*	intron		C/G	0.21		ACGTTGGATGGGAACCGAGTAAGGAAGGAC	ACGTTGGATGCACCCTTCACCACATCCCAT	GGAAGGACTCGTGCATTA
1	rs3775291	4	187004074	*TLR3*	exon 4	missense	G/A	0.33		ACGTTGGATGTATCACTTGCTCATTCTCCC	ACGTTGGATGGAGAAAGCATCACTCTCTAT	TGCTCATTCTCCCTTACACATA
1	rs2770150	9	120463139	*TLR4*	near-gene-5		T/C	0.32		ACGTTGGATGCACTCAATCATTTACTGACC	ACGTTGGATGACACATGGTCTGCCTTCTGG	ATCAAAGACATCTAGGTTCTATG
1	rs1927914	9	120464725	*TLR4*	near-gene-5		T/C	0.31		ACGTTGGATGGTGCTTGGAGGATATTACAG	ACGTTGGATGGAACCTGATTTAAAACAGGA	GAACTATCTAGGACTTAGCAT
1	rs1927911	9	120470054	*TLR4*	intron		C/T	0.25		ACGTTGGATGAGACCTTCCTTAGTCATGGC	ACGTTGGATGCATCACTTTGCTCAAGGGTC	CCAGATTTTGACAACTGCATTCTTTT
1	rs10759933	9	120470372	*TLR4*	intron		A/C	-		ACGTTGGATGGGTAATAAAATATCCAATATCG	ACGTTGGATGTGCTCATCTTCTCTGTATCC	AAAATATCCAATATCGTGCTTGC
1	rs1927907	9	120472764	*TLR4*	intron		G/A	0.14		ACGTTGGATGTTTTTCAAACAAGAAGTAG	ACGTTGGATGGGGTATCCAGTGGATTGAAG	TCAAACAAGAAGTAGTTTTTCA
1	rs7869402	9	120478032	*TLR4*	exon 3	UTR-3	C/T	0.03		ACGTTGGATGTTTAGGGAGACACAGATGGC	ACGTTGGATGACCTTCACACGTAGTTCTCC	TGGCTGGGATCCCTCCCCTGTACC
1	rs7873784	9	120478936	*TLR4*	exon 4	UTR-3	G/C	0.14		ACGTTGGATGGCTCTAAAGATCAGCTGTAT	ACGTTGGATGGGTACCCTCTTAACAAAATG	AAGATCAGCTGTATAGCAGAGTTC

1	rs2302267	X	12885578	*TLR7*	intron		T/G	0.03		ACGTTGGATGAGCTACAGTATTGTGCTGTC	ACGTTGGATGATGGGCCCAATAGCATCAAC	TTGTGCTGTCTTTGAAATGTAAACTT
1	rs5741880	X	12887416	*TLR7*	intron		G/T	0.13		ACGTTGGATGGTGTAATCTCTAGATCAAAG	ACGTTGGATGAAAGGCAAAAAAGCACAGGG	TCTCTAGATCAAAGGATCTG
1	rs179022	X	12888567	*TLR7*	intron		G/T	-		ACGTTGGATGTAGGAAATTTCTTTATGGC	ACGTTGGATGCCAAATTTTTAAATAATCAG	GGCTAAAAAAAGGTTATTAAGTAATC
1,2,3	rs179021	X	12889763	*TLR7*	intron		A/C	0.21	0.00	ACGTTGGATGGGCTCAAGCAACTCTTTGTC	ACGTTGGATGCAGAAAAAAGCCAAGTTGCC	CAGATCCCAACATTTCCTTT
1,2,3	rs179020	X	12889857	*TLR7*	intron		C/T	-	-	ACGTTGGATGATGGGGCTGTTGTCCTCTC	ACGTTGGATGCTAACCAACCAGTCAGAAAG	GGCTGTTGTCCTCTCAGGGCT
1,2,3	rs179019	X	12889969	*TLR7*	intron		G/T	0.21	0.30	ACGTTGGATGGAGATGAGACTATTTGAGGC	ACGTTGGATGAGTCAGGAGAGCAGGAAATC	TGAGGCCTGGGGAACGCTT
1,2,3	rs179017	X	12893793	*TLR7*	intron		G/T	-	-	ACGTTGGATGGTCTCACAAGATCTGATGG	ACGTTGGATGATTACGTCTTACATGGCGTC	ACAAGATCTGATGGTTTCATAAACGGC
1,2,3	rs179016	X	12894442	*TLR7*	intron		C/G	0.34	0.14	ACGTTGGATGAACCAATTTCTCTATGCCCC	ACGTTGGATGGTTCAAGTATTCTTGGTACTC	ATTTCTCTATGCCCCAAAACC
1	rs1634321	X	12895325	*TLR7*	intron		T/A	0.10		ACGTTGGATGACTTTCTCTTTAAATAACAG	ACGTTGGATGAGGCTGTGTTAAACTTGTG	CTTTCTCTTTAAATAACAGATTTAGA
2,3	rs179014	X	12899765	*TLR7*	intron		C/T	0.21	0.02	ACGTTGGATGAATAACTATTGAGGCCTGGG	ACGTTGGATGAGAGTGGACAATCCTATATG	TTCCATAATCAAACACACAAAATTTC
2,3	rs179013	X	12901471	*TLR7*	intron		C/T	0.20	0.00	ACGTTGGATGAAGTAGAAATCTGGTGGGTG	ACGTTGGATGACCCAAGTTCCCTGACTTGC	aagaTGGTGGGTGAAAATGGTAG
2,3	rs179012	X	12901562	*TLR7*	intron		C/T	0.27	0.08	ACGTTGGATGCATGGCATCCAAAGTCTTCC	ACGTTGGATGGTGAGGCTAGTAGAGGAATT	gtcATTTGTACCCTGTCTACTTGTC
1,2,3	rs179011	X	12901960	*TLR7*	intron		C/A	0.20	0.00	ACGTTGGATGGAGGTACAAAATTGTCTCTTC	ACGTTGGATGCAGCTGGACTCTCAATGTTT	AATTGTCTCTTCTTCTTGTCACC
2,3	rs179010	X	12902885	*TLR7*	intron		C/T	0.26	0.34	ACGTTGGATGTGGATCAGTTATACGGTTCC	ACGTTGGATGTGGAGAGGAGATAGTAGTAG	ggTCCTCTGATTATGTATCACC
1,2,3	rs179008	X	12903659	*TLR7*	exon 3	missense	A/T	0.20	0.00	ACGTTGGATGATCTAGCCCCAAGGAGTTTG	ACGTTGGATGGTTTCCAATGTGGACACTGA	GGATTATGTTAAAAAGGATAAGAATT
1,2,3	rs864058	X	12906030	*TLR7*	exon 3	cds-synonymous	C/T	0.12	0.02	ACGTTGGATGCACACAAGTCACATCTGTGG	ACGTTGGATGGTGATGCTGTGTGGTTTGTC	GTGGCCAGGTAAGGAATAGTCACCTC
1	rs3853839	X	12907658	*TLR7*	exon 3	UTR-3	T/C	0.00		ACGTTGGATGCTTTCTTTCTTACTGTTTCCC	ACGTTGGATGGGTTGCTGCTTCAGTGCTTC	CAGAAGCAGGCCCAAG
2,3	rs179007	X	12910322	*TLR7*	near-gene-3		T/C	0.34	0.02	ACGTTGGATGTTTCTTCTTTTGCCTCCCCC	ACGTTGGATGTTGCTTGACACTTGCTGTGG	ctgaCTCCCCCTCCCTCTC
2,3	rs179006	X	12910521	*TLR7*	near-gene-3		G/A	0.33	0.00	ACGTTGGATGTGAATGTGTCAGAGTCCAGC	ACGTTGGATGCTTCAGTGAGCATGATCTTC	gaacGTGTCACCTAGGGAAGATTCCA
2,3	rs2269809	X	12910619	*TLR7*	near-gene-3		A/C	0.44	0.18	ACGTTGGATGCCACTGATGTCATGACAAAC	ACGTTGGATGCTTTCAAGCACTAATTCAGTC	taccACCTGAGATTAGGGGA
2,3	rs5935438	X	12913022	*TLR7*	near-gene-3		G/C	0.42	0.14	ACGTTGGATGTGACTTAATTAACGGGCGTG	ACGTTGGATGGCCTACTTGACTGTCAGTTC	GCATCATGCCTGTTCA
2,3	rs178998	X	12917787	*TLR8*	near-gene-5		G/A	0.28	0.86	ACGTTGGATGCCTAAACTGTATGCATTGCC	ACGTTGGATGTGGGCCTTCCTGTAGAAGAC	gggtGGGGAAGCAACCTAGT
2,3	rs3788935	X	12922659	*TLR8*	near-gene-5		A/G	0.22	0.81	ACGTTGGATGGTCTAGTATCTATGTCAAGC	ACGTTGGATGGTGTGGAGAAAACTGAAGGC	AACTCATAAAAATGAGTTACTTACTTA
1,2,3	rs3761624	X	12923681	*TLR8*	near-gene-5		A/G	0.22	0.83	ACGTTGGATGTTGGTTTTCTCCCACTCCTG	ACGTTGGATGCCCTGGCCACAAGAATAAAG	GTAAGGCAAGATGAAACAT
2,3	rs5741883	X	12924221	*TLR8*	near-gene-5		C/T	0.23	0.05	ACGTTGGATGAAGCGAGCATCTTTCTCCTG	ACGTTGGATGACAATGAACACTCATTGAGC	gaaaATGCCTCCTCCAGCACCTGGC
1	rs3764880	X	12924826	*TLR8*	exon 1	missense	A/G	0.22		ACGTTGGATGGCTAAAGAAATAGAAGTGGC	ACGTTGGATGCTGCTGCAAGTTACGGAATG	GAAATAGAAGTGGCTTACCA
2,3	rs17256081	X	12926045	*TLR8*	intron		C/T	0.48	0.89	ACGTTGGATGGCACTAAAATTTTACAATGC	ACGTTGGATGATGTGTCATTGGCCCAGTTG	GCTTTACAAAATGACTGTAGG
1	rs2109134	X	12927186	*TLR8*	intron		A/T	0.08		ACGTTGGATGCCCAGATGAACTCAGAGATG	ACGTTGGATGAAGTGCATCATAGTGTCGCC	cATGACGTTCCCCTCAATGGA
1,2,3	rs4830805	X	12927759	*TLR8*	intron		G/A	0.20	0.81	ACGTTGGATGAAGGGAACGTGGAAAATCCG	ACGTTGGATGATCAGCAGAGACCTGATAGC	ACCAGCATTTGAGTCTTGGA
1,2,3	rs1548731	X	12927947	*TLR8*	intron		C/T	0.23	0.05	ACGTTGGATGACCCAAAGAAGTCCATGAGG	ACGTTGGATGGAGTGACCTTGAAAACATTC	TCCATGAGGCCCCTGCTTT
1,2,3	rs4830808	X	12932334	*TLR8*	intron		C/T	0.16	0.73	ACGTTGGATGGGTAAATGGGTCTCCATTTAG	ACGTTGGATGTCCCACTGGAATTGTTTAGG	TAGAAAAAAATAATGTGGGTCTA
1	rs1013150	X	12932441	*TLR8*	intron		G/A	0.19		ACGTTGGATGATCACTCAGGGCAGTGTAAG	ACGTTGGATGTTACTAGGAAAAAGATGAG	CAGGGCAGTGTAAGGCATAAAGGAT
1	rs5744068	X	12935058	*TLR8*	intron		C/T	0.17		ACGTTGGATGTCATTTCCTTGCTAGCTGCC	ACGTTGGATGCCCACAACAAGGAATGCAAG	CCTGCAGAGAGCCACTCTCAGCTTCCA
1	rs5744080	X	12937804	*TLR8*	exon 2	cds-synonymous	C/T	0.34		ACGTTGGATGGCTGACAAATTTGGAGTTGC	ACGTTGGATGGTAGGGAGCTTGGCAGTTTG	TATCTTTCAATTCTCTTTCACA
2,3	rs2407992	X	12939112	*TLR8*	exon 2	cds-synonymous	G/C	0.34	0.83	ACGTTGGATGGACTCGCTGGCAAATTAAGG	ACGTTGGATGGACACGTCTGGATTTATCCC	GCTTCATTTGGGATGTGCTT
1	rs3747414	X	12939412	*TLR8*	exon 2	cds-synonymous	C/A	0.31		ACGTTGGATGGGTCTTAGTTTCAAGTGCGG	ACGTTGGATGAGTCAGTAGTCTGAAGCACC	TCAAGTGCGGATTTGTT

*1, Swedish population, first screen; 2, Swedish population, second screen; 3, Chinese population.
