# Supplementary material for: Toll-like receptor gene polymorphisms are associated with allergic rhinitis: a case control study
Source: BMC Med Genet. 2012 Aug 2;13:66. doi: 10.1186/1471-2350-13-66 (PMC3459792; doi:10.1186/1471-2350-13-66)
Supplement: Additional file 3 — Description of genotyping and association testing in the two populations. [file 1471-2350-13-66-S3.pdf]

Text S1. Description of genotyping and association testing in the two populations.

Genotyping

All reactions were run under the same conditions except for the primer annealing temperature of the primary PCR. PCR reactions were performed in a total volume of 6 µl containing 2.5 ng of template DNA, 1.25X HotStar Taq PCR buffer, 0.15 units of HotStar Taq polymerase, 3.5 mM $MgCl_2$, 0.5 mM dNTPs and 100 nM of each primer. Amplifications were performed using GeneAmp 9700 machines with dual-384 heads as follows: 95°C for 15 min, 45 cycles of 95°C for 20 s, 56°C, 60°C or 64°C for 30 s, 72°C for 60 s and finally 72°C for 3 min. Dephosphorylation of unincorporated dNTPs was achieved using shrimp alkaline phosphatase. Concentrations of individual hME primer pairs were adjusted to even out peak heights in the mass spectrum. The extension reactions were then made by mixing the adjusted MassEXTEND primer mix (containing approximately 1 µM of each primer) with hME EXTEND mix containing buffer and 50 µM of each d/ddNTP mix and 1.25 units of Thermo Sequenase. PCR amplification of hME reactions was performed as follows: 94°C for 2 min and 99 cycles of 94°C for 5 s, 52°C for 5 s and 72°C for 5 s. The samples were then manually desalted by using 6 mg of Clean Resin and a dimple plate and subsequently transferred to a 384-well SpectroCHIP using a nanodispenser.

Association testing *TLR1-TLR10* in the Swedish population

Of the 107 SNPs initially attempted, 73 were successfully genotyped; 21 did not produce a working assay, 3 failed the genotyping cut-off of 95% and 10 were monomorphic in the study population. Since none of the assays for *TLR5* worked due to multiple hits elsewhere in the genome, this gene was excluded from further analysis. The total genotyping rate after removal of low-quality SNPs and DNA samples was 99.1% in 182 cases and 378 controls. All SNPs attempted and successfully genotyped are summarized in Tables S1 and S2. The SNPs were genotyped in a subset of the Swedish population because this genotyping was completed before the collection of the additional Swedish individuals and before establishing collaboration with the Singapore group.

Association testing the *TLR7-TLR8* region in the Swedish population

Based on the results obtained in the first experiment, additional SNPs covering the *TLR7-TLR8* region were selected together with two SNPs reported in the literature as being associated with AR (rs179008 and rs2407992; Møller-Larsen *et al.* [12]) and genotyped in the

complete Swedish population consisting of 360 cases and 720 controls. Of the 30 SNPs initially attempted, 24 were successfully genotyped; 1 did not produce a working assay and 5 failed the genotyping cutoff of 95%. All SNPs attempted and successfully genotyped are summarized in Tables S1 and S2. Samples of low quality were excluded from further analysis and the total genotyping rate after removal of low-quality data was 99.5%. This set of 24 SNPs were then analyzed for association with AR in 352 (168 female, 184 male) cases and 709 (291 female, 418 male) controls. Haplotypes were constructed based on the significantly associated SNPs and subsequently tested for association.

Association testing the *TLR7-TLR8* region in the Chinese population

The same set of SNPs as in the second experiment was genotyped in a Chinese population consisting of 1024 AR cases and 605 controls. Of the 30 SNPs initially attempted, 23 were successfully genotyped; 1 did not produce a working assay and 6 failed the genotyping cut off of 95%. Samples of low quality were excluded from further analysis and the total genotyping rate after removal of low-quality data was 99.7%. The remaining 23 SNPs were then analyzed for association with AR in 948 (514 female, 434 male) cases and 580 (440 female, 140 male) controls. All SNPs attempted and successfully genotyped are summarized in Tables S1 and S2. Haplotypes were constructed based on the significantly associated SNPs and subsequently tested for association.
