# Supplementary material for: Toll-like receptor gene polymorphisms are associated with allergic rhinitis: a case control study
Source: BMC Med Genet. 2012 Aug 2;13:66. doi: 10.1186/1471-2350-13-66 (PMC3459792; doi:10.1186/1471-2350-13-66)
Supplement: Additional file 4 — Complete first experiment association test results in the Swedish population. [file 1471-2350-13-66-S4.pdf]

Table S3. Complete first experiment association test results in the Swedish population

P -values are given for the Hardy-Weinberg (HW) and association tests.
HW test uses cases and controls. Q-values for the FDR-test are given in paranthesis.

Gene	SNP ID	Chromosome position		Study MAF	HW test	Association test	
						Allele	Genotype
TLR9	rs352140	3	52256697	0.45	0.92	0.59	0.73
TLR9	rs352139	3	52258372	0.43	0.76	0.48	0.70
TLR9	rs5743836	3	52260782	0.13	0.28	0.74	0.87
TLR9	rs187084	3	52261031	0.44	0.93	0.68	0.28
TLR10	rs10776483	4	38775040	0.27	0.75	0.44	0.54
TLR10	rs11096955	4	38776107	0.42	1.0	0.32	0.46
TLR10	rs11096956	4	38776180	0.26	0.57	0.47	0.56
TLR10	rs11096957	4	38776491	0.42	0.97	0.35	0.49
TLR10	rs10856839	4	38777236	0.19	0.11	0.71	0.82
TLR10	rs4274855	4	38777471	0.22	0.62	0.55	0.22
TLR10	rs7694115	4	38779094	0.41	0.62	0.39	0.64
TLR10	rs7698870	4	38781459	0.04	0.30	0.70	-
TLR1	rs4833095	4	38799710	0.30	0.83	0.046 (0.29)	0.056
TLR1	rs5743596	4	38802528	0.20	0.89	0.85	0.50
TLR1	rs5743595	4	38802644	0.22	0.68	0.41	0.38
TLR1	rs5743594	4	38802751	0.17	0.35	0.096	0.26
TLR1	rs5743580	4	38804405	0.22	0.68	0.36	0.33
TLR1	rs5743566	4	38805942	0.22	0.63	0.51	0.51
TLR1	rs5743565	4	38805983	0.22	0.65	0.42	0.40
TLR1	rs5743557	4	38806827	0.23	0.76	0.44	0.41
TLR6	rs5743818	4	38829163	0.26	0.81	0.39	0.27
TLR6	rs3821985	4	38830012	0.34	0.57	0.76	0.45
TLR6	rs5743810	4	38830350	0.37	0.38	0.29	0.18
TLR6	rs5743808	4	38830736	0.00	0.95	0.23	-
TLR6	rs1039559	4	38831596	0.43	0.036	0.30	0.16
TLR6	rs5743805	4	38832059	0.03	0.23	0.046 (0.29)	0.15
TLR6	rs5743788	4	38833207	0.48	0.055	0.35	0.30
TLR2	rs893629	4	154604968	0.01	0.90	0.40	-
TLR2	rs4696480	4	154607126	0.46	0.96	0.19	0.43
TLR2	rs1898830	4	154608453	0.31	0.31	0.79	0.39
TLR2	rs1816702	4	154609523	0.10	0.16	0.072	0.18
TLR2	rs4235232	4	154618084	0.01	0.90	0.41	-
TLR2	rs3804099	4	154624656	0.43	0.43	0.35	0.28
TLR2	rs3804100	4	154625409	0.08	0.20	0.72	0.93
TLR2	rs5743704	4	154625951	0.04	0.69	0.56	0.73

TLR3	rs5743303	4	186988853	0.19	0.76	0.58	0.86
TLR3	rs5743305	4	186989333	0.34	0.66	0.095	0.081
TLR3	rs7657186	4	186994039	0.18	0.86	0.30	0.49
TLR3	rs3775296	4	186997767	0.20	0.92	0.67	0.90
TLR3	rs5743312	4	187000256	0.16	0.79	0.76	0.90
TLR3	rs5743314	4	187000375	0.19	0.47	0.37	0.60
TLR3	rs3775292	4	187003025	0.18	0.19	0.23	0.29
TLR3	rs3775291	4	187004074	0.29	0.19	0.16	0.32
TLR4	rs2770150	9	120463139	0.25	0.85	0.55	0.84
TLR4	rs1927914	9	120464725	0.34	0.99	0.27	0.50
TLR4	rs1927911	9	120470054	0.28	0.26	0.76	0.57
TLR4	rs10759933	9	120470372	0.04	0.88	0.29	0.28
TLR4	rs1927907	9	120472764	0.15	0.24	0.72	0.73
TLR4	rs7869402	9	120478032	0.03	0.65	0.81	0.70
TLR4	rs7873784	9	120478936	0.17	0.58	0.88	0.95
TLR7	rs2302267	X	12885578	0.03	0.76	0.62	0.35
TLR7	rs5741880	X	12887416	0.07	0.80	0.097	0.41
TLR7	rs179022	X	12888567	0.40	0.81	0.83	0.025 (0.47)
TLR7	rs179021	X	12889763	0.20	0.78	0.16	0.31
TLR7	rs179020	X	12889857	0.25	0.59	0.13	0.37
TLR7	rs179019	X	12889969	0.25	0.56	0.18	0.45
TLR7	rs179017	X	12893793	0.09	0.60	0.92	0.50
TLR7	rs179016	X	12894442	0.33	0.48	0.19	0.46
TLR7	rs1634321	X	12895325	0.07	0.82	0.68	0.56
TLR7	rs179011	X	12901960	0.20	0.59	0.31	0.56
TLR7	rs179008	X	12903659	0.20	0.90	0.23	0.32
TLR7	rs864058	X	12906030	0.06	0.34	0.30	-
TLR7	rs3853839	X	12907658	0.16	0.96	0.90	0.40
TLR8	rs3761624	X	12923681	0.21	0.97	0.043 (0.29)	0.13
TLR8	rs3764880	X	12924826	0.21	0.98	0.034 (0.29)	0.12
TLR8	rs2109134	X	12927186	0.07	0.24	0.40	-
TLR8	rs4830805	X	12927759	0.19	0.48	0.017 (0.29)	0.12
TLR8	rs1548731	X	12927947	0.25	0.70	0.49	0.058
TLR8	rs4830808	X	12932334	0.16	0.67	0.93	0.69
TLR8	rs1013150	X	12932441	0.20	0.76	0.21	0.65
TLR8	rs5744068	X	12935058	0.16	0.44	0.27	0.78
TLR8	rs5744080	X	12937804	0.37	0.026	0.73	0.69
TLR8	rs3747414	X	12939412	0.32	0.070	0.76	0.83
