# Supplementary material for: Toll-like receptor gene polymorphisms are associated with allergic rhinitis: a case control study
Source: BMC Med Genet. 2012 Aug 2;13:66. doi: 10.1186/1471-2350-13-66 (PMC3459792; doi:10.1186/1471-2350-13-66)
Supplement: Additional file 5 — Allele and genotype frequencies for all significant SNPs in the Swedish population. [file 1471-2350-13-66-S5.pdf]

Table S4. Allele and genotype frequencies for all significant SNPs in the Swedish population

Odds ratios (OR) and 95% confidence intervals (CI) are calculated for the risk allele.

SNP ID	Allele/Genotype	Allele/Genotype frequency		OR	95% CI	
		Controls	Cases		Lower	Upper
rs2269809	G	0.49	0.52	1.10	0.89	1.36
	T	0.51	0.48	-	-	-
	GG	0.22	0.32	1.19	0.68	2.08
	GT	0.59	0.46	0.66	0.40	1.09
	TT	0.19	0.22	-	-	-
rs5935438	C	0.44	0.48	1.16	0.94	1.44
	G	0.56	0.52	-	-	-
	CC	0.16	0.28	1.66	0.96	2.90
	GC	0.58	0.45	0.75	0.47	1.20
	GG	0.26	0.27	-	-	-
rs3788935	A	0.75	0.82	1.48	1.14	1.93
	G	0.25	0.18	-	-	-
	AA	0.59	0.70	4.35	1.44	19.54
	AG	0.34	0.28	3.02	0.97	13.88
	GG	0.07	0.02	-	-	-
rs3761624	A	0.76	0.82	1.56	1.20	2.04
	G	0.24	0.18	-	-	-
	AA	0.59	0.69	9.31	3.08	41.85
	AG	0.34	0.29	3.02	0.97	13.88
	GG	0.07	0.02	-	-	-
rs17256081	C	0.47	0.52	1.18	0.95	1.46
	T	0.53	0.48	-	-	-
	CC	0.18	0.29	1.57	0.90	2.73
	CT	0.58	0.47	0.80	0.50	1.30
	TT	0.24	0.24	-	-	-
rs4830805	G	0.77	0.83	1.45	1.11	1.92
	A	0.23	0.17	-	-	-
	GG	0.61	0.71	3.22	1.02	14.76
	GA	0.34	0.27	2.23	0.68	10.43
	AA	0.05	0.02	-	-	-
rs1548731	T	0.27	0.28	1.08	0.85	1.37
	C	0.74	0.72	-	-	-
	TT	0.05	0.11	1.98	0.97	4.12
	CT	0.43	0.35	0.80	0.53	1.20
	CC	0.52	0.54	-	-	-
