# Supplementary material for: Toll-like receptor gene polymorphisms are associated with allergic rhinitis: a case control study
Source: BMC Med Genet. 2012 Aug 2;13:66. doi: 10.1186/1471-2350-13-66 (PMC3459792; doi:10.1186/1471-2350-13-66)
Supplement: Additional file 6 — Linkage disequilibrium plot of TLR7-TLR8 gene region constructed using Haploview software and HapMap data (release 24) of the A) CEU population and B) CHB population. The intensity of shading represents D´. S SNPs showing single-point association in the Swedish population. C SNPs showing single-point association in the Chinese population. D SNPs showing single-point association in Møller-Larsen et al. (2008). [file 1471-2350-13-66-S6.pdf]

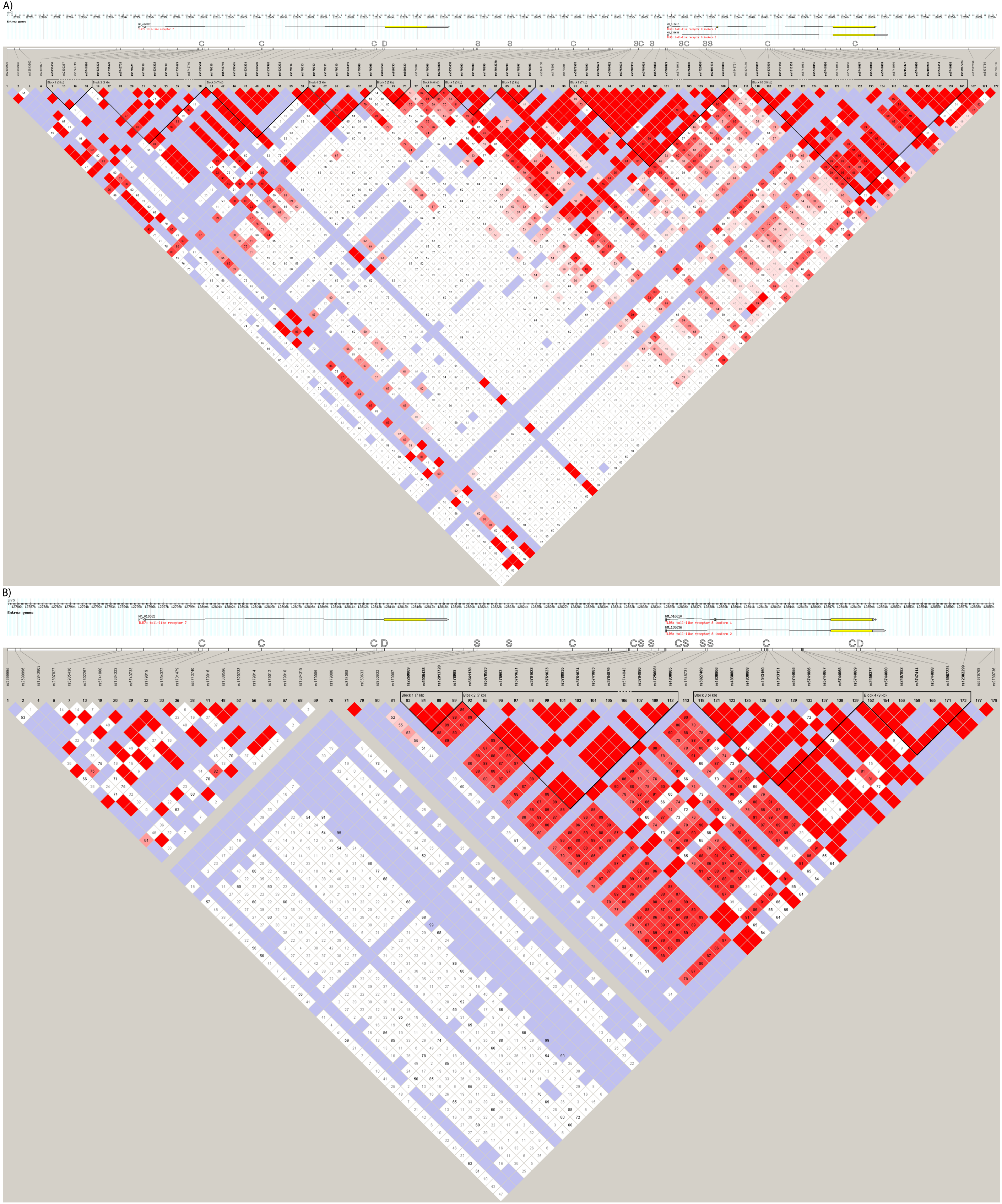

Figure S1. Linkage disequilibrium plot of TLR7-TLR8 gene region constructed using Haploview software and HapMap data (release 24) of the A) CEU population and B) CHB population. The intensity of shading represents D´.[S] SNPs showing single-point association in the Swedish population. [C] SNPs showing single-point association in the Chinese population. [D] SNPs showing single-point association in Møller-Larsen et al. (2008)
