# Supplementary material for: Toll-like receptor gene polymorphisms are associated with allergic rhinitis: a case control study
Source: BMC Med Genet. 2012 Aug 2;13:66. doi: 10.1186/1471-2350-13-66 (PMC3459792; doi:10.1186/1471-2350-13-66)
Supplement: Additional file 7 — Allele frequencies in Chinese males for all significant SNPs [file 1471-2350-13-66-S7.pdf]

Table S5. Allele frequencies in Chinese males for all significant SNPs

Odds ratios (OR) and 95% confidence intervals (CI) are calculated for the risk allele.

SNP ID	Allele	Allele frequency		OR	95% CI	
		Controls	Cases		Lower	Upper
rs178998	T	0.79	0.87	1.77	1.06	2.89
	C	0.21	0.13	-	-	-
rs3788935	G	0.77	0.85	1.71	1.05	2.75
	A	0.23	0.15	-	-	-
rs17256081	T	0.79	0.88	1.92	1.15	3.16
	C	0.21	0.12	-	-	-
rs4830805	A	0.74	0.83	1.74	1.09	2.74
	G	0.26	0.17	-	-	-
rs2407992	C	0.75	0.85	1.92	1.20	3.05
	G	0.25	0.15	-	-	-
